# Supplementary material for: MRI-based deep learning can discriminate between temporal lobe epilepsy, Alzheimer’s disease, and healthy controls
Source: Commun Med (Lond). 2023 Feb 27;3:33. doi: 10.1038/s43856-023-00262-4 (PMC9970972; doi:10.1038/s43856-023-00262-4)
Supplement: Supplementary file 2 — Supplementary Information [file 43856_2023_262_MOESM2_ESM.pdf]

ADNI Alzheimer's Disease Neuroimaging Initiative

ACKNOWLEDGEMENT LIST FOR ADNI PUBLICATIONS

The Data and Publications Committee, in keeping with the publication policies adopted by the ADNI Steering Committee, here provide lists for standardized acknowledgement. The list consists of three parts: I. ADNI Infrastructure Investigators and Site Investigators, II. DOD ADNI Infrastructure Investigators and Site Investigators and III. ADNI Depression Infrastructure Investigators and Site Investigators. Infrastructure Investigators represent the names responsible for leadership and infrastructure. Site Investigators represent the names of individuals at each recruiting site. All papers, including methodological papers, should have an acknowledgement list that consists of Infrastructure Investigators plus the FULL list.

I. ADNI I, GO, II and III

Part A: Leadership and Infrastructure

Principal Investigator

Michael W. Weiner, MD	University of California, San Francisco

ATRI PI and Director of Coordinating Center Clinical Core

Paul Aisen, MD	University of Southern California
Co PI of Clinical Core Ronald Petersen, MD, PhD	Mayo Clinic, Rochester

Executive Committee

Michael W. Weiner, MD	University of California, San Francisco
Paul Aisen, MD	University of Southern California
Ronald Petersen, MD, PhD	Mayo Clinic, Rochester
Clifford R. Jack, Jr., MD	Mayo Clinic, Rochester
William Jagust, MD	University of California, Berkeley
John Q. Trojanowki, MD, PhD	University of Pennsylvania
Arthur W. Toga, PhD	University of Southern California
Laurel Beckett, PhD	University of California, Davis
Robert C. Green, MD, MPH	Brigham and Women's Hospital/Harvard Medical School
Andrew J. Saykin, PsyD	Indiana University
John C. Morris, MD	Washington University St. Louis
Richard J. Perrin, MD, PhD	Washington University St. Louis
Leslie M. Shaw, PhD	University of Pennsylvania

ADNI External Advisory Board (ESAB)

Zaven Khachaturian, PhD	Prevent Alzheimer's Disease 2020 (Chair)
Maria Carrillo, PhD	Alzheimer's Association
William Potter, MD	National Institute of Mental Health
Lisa Barnes, PhD	Rush University
Marie Bernard, MD	NIA
Hector González	University of California, San Diego
Carole Ho	Denali Therapeutics
John K. Hsiao, MD	NIH
Jonathan Jackson, PhD	Massachusetts General Hospital
Eliezer Masliah, MD	NIA
Donna Masterman, MD	Biogen
Ozioma Okonkwo, PhD	University of Wisconsin, Madison

Richard Perrin, MD	Washington University
Laurie Ryan, PhD	NIA
Nina Silverberg, PhD	NIA

ADNI 3 Private Partner Scientific Board (PPSB)

Adam Fleisher, MD	Eli Lilly (Chair)

Administrative Core - Northern California Institute for Research & Education (NCIRE / The Vererans Health Research Institute)

Michael W. Weiner, MD	University of California, San Francisco
Diana Truran Sacrey,	NCIRE / The Vererans Health Research Institute
Juliet Fockler,	University of California, San Francisco
Cat Conti, BA	NCIRE / The Vererans Health Research Institute
Dallas Veitch, PhD	NCIRE / The Vererans Health Research Institute
John Neuhaus, PhD	University of California, San Francisco
Chengshi Jin, PhD	University of California, San Francisco
Rachel Nosheny, PhD	University of California, San Francisco
Miriam Ashford, PhD	NCIRE / The Vererans Health Research Institute
Derek Flenniken,	NCIRE / The Vererans Health Research Institute
Adrienne Kormos,	NCIRE / The Vererans Health Research Institute

Data and Publications Committee

Robert C. Green, MD, MPH	BWH/HMS (Chair)

Resource Allocation Review Committee

Tom Montine, MD, PhD	University of Washington (Chair)
Cat Conti, BA	NCIRE / The Vererans Health Research Institute

Clinical Core Leaders and Key Personnel

Ronald Petersen, MD, PhD	Mayo Clinic, Rochester (Core PI)
Paul Aisen, MD	University of Southern California (Core PI)
Michael Rafii, MD, PhD	University of Southern California
Rema Raman, PhD	University of Southern California
Gustavo Jimenez, MBS	University of Southern California
Michael Donohue, PhD	University of Southern California
Devon Gessert, BS	University of Southern California
Jennifer Salazar, MBS	University of Southern California
Caileigh Zimmerman, MS	University of Southern California
Yuliana Cabrera, BS	University of Southern California
Sarah Walter, MSc	University of Southern California
Garrett Miller, MS	University of Southern California
Godfrey Coker, MBA, MPH	University of Southern California
Taylor Clanton, MPH	University of Southern California
Lindsey Hergesheimer, BS	University of Southern California
Stephanie Smith, BS	University of Southern California
Olusegun Adegoke, MSc	University of Southern California
Payam Mahboubi, MPH	University of Southern California
Shelley Moore, BA	University of Southern California
Jeremy Pizzola, BA	University of Southern California
Elizabeth Shaffer, BS	University of Southern California

ADNI Alzheimer's Disease Neuroimaging Initiative

Brittany Sloan, BA
University of Southern California

Biostatistics Core Leaders and Key Personnel

Laurel Beckett, PhD	University of California, Davis (Core PI)
Danielle Harvey, PhD	University of California, Davis
Michael Donohue, PhD	University of Southern California

MRI Core Leaders and Key Personnel

Clifford R. Jack, Jr., MD	Mayo Clinic, Rochester (Core PI)
Arvin Forghanian-Arani, PhD	Mayo Clinic
Bret Borowski, RTR	Mayo Clinic
Chad Ward,	Mayo Clinic
Christopher Schwarz, PhD	Mayo Clinic
David Jones, MD	Mayo Clinic
Jeff Gunter, PhD	Mayo Clinic
Kejal Kantarci, MD	Mayo Clinic
Matthew Senjem, MS	Mayo Clinic
Prashanthi Vemuri, PhD	Mayo Clinic
Robert Reid, PhD	Mayo Clinic
Nick C. Fox, MD	University College London
Ian Malone, PhD	University College London
Paul Thompson, PhD	University of Southern California School of Medicine
Sophia I. Thomopoulos, BS	University of Southern California School of Medicine
Talia M. Nir, PhD	University of Southern California School of Medicine
Neda Jahanshad, PhD	University of Southern California School of Medicine
Charles DeCarli, MD	University of California, Davis
Alexander Knaack, MS	University of California, Davis
Evan Fletcher, PhD	University of California, Davis
Danielle Harvey, PhD	University of California, Davis
Duygu Tosun-Turgut, PhD	University of California, San Francisco
Stephanie Rossi Chen, BA.	NCIRE / The Vererans Health Research Institute
Mark Choe, BS	NCIRE / The Vererans Health Research Institute
Karen Crawford,	University of Southern California School of Medicine
Paul A. Yushkevich, PhD	University of Pennsylvania
Sandhitsu Das, PhD	University of Pennsylvania

PET Core Leaders and Key Personnel

William Jagust, MD	University of California, Berkeley (Core PI)
Robert A. Koeppe, PhD	University of Michigan
Eric M. Reiman, MD	Banner Alzheimer's Institute
Kewei Chen, PhD	Banner Alzheimer's Institute
Chet Mathis, MD	University of Pittsburgh
Susan Landau, PhD	University of California, Berkeley

Neuropathology Core Leaders and Key Personnel

John C. Morris, MD	Washington University St. Louis
Richard Perrin MD	Washington University St. Louis
Nigel J. Cairns, PhD, FRCPath	Washington University St. Louis—Past Investigator
Erin Householder, MS	Washington University St. Louis

Erin Franklin, MS — Washington University St. Louis
Haley Bernhardt, BA, R. EEG T — Washington University St. Louis
Lisa Taylor-Reinwald, BA, HTL (ASCP) – Past Investigator — Washington University St. Louis

Biomarkers Core Leaders and Key Personnel

Leslie M. Shaw, PhD	Perelman School of Medicine, University of Pennsylvania (co-PI)
John Q. Trojanowki, MD, PhD	Perelman School of Medicine, University of Pennsylvania (co-PI)
Magdalena Korecka, PhD	Perelman School of Medicine, University of Pennsylvania
Michal Figurski, PhD	Perelman School of Medicine, University of Pennsylvania

Informatics Core Leaders and Key Personnel

Arthur W. Toga, PhD	University of Southern California (Core PI)
Karen Crawford	University of Southern California
Scott Neu, PhD	University of Southern California

Genetics Core Leaders and Key Personnel

Andrew J. Saykin, PsyD	Indiana University School of Medicine (Core PI)
Kwangsik Nho, PhD	Indiana University School of Medicine
Shannon L. Risacher, PhD	Indiana University School of Medicine
Liana G. Apostolova, MD	Indiana University School of Medicine
Li Shen, PhD	UPenn School of Medicine
Tatiana M. Foroud, PhD	NCRAD/Indiana University School of Medicine
Kelly Nudelman, PhD	NCRAD/Indiana University School of Medicine
Kelley Faber, MS, CCRC	NCRAD/Indiana University School of Medicine
Kristi Wilmes, MS, CCRP	NCRAD/Indiana University School of Medicine

Initial Concept Planning & Development

Michael W. Weiner, MD	University of California, San Francisco
Leon Thal, MD – Past Investigator	University of California, San Diego
Zaven Khachaturian, PhD	Prevent Alzheimer's Disease 2020

NIA

John K. Hsiao, MD	National Institute on Aging

<u>Part B: Investigators By Site</u>

Oregon Health & Science University:
Lisa C. Silbert, MD
Betty Lind, BS
Rachel Crissey
Jeffrey A. Kaye, MD, A – Past Investigator
Raina Carter, BA – Past Investigator
Sara Dolen, BS – Past Investigator
Joseph Quinn, MD – Past Investigator

University of Southern California:
Lon S. Schneider, MD
Sonia Pawluczyk, MD
Mauricio Becerra, MD
Liberty Teodoro, RN
Karen Dagerman, MS
Bryan M. Spann, DO, PhD – Past Investigator

University of California – San Diego:
James Brewer, MD, PhD
Helen Vanderswag, RN

Adam Fleisher, MD – Past Investigator

University of Michigan:
Jaimie Ziolkowski, MA, BS, TLLP
Judith L. Heidebrink, MD, MS
Lisa Zbizek-Nulph, MS
Joanne L. Lord, LPN, BA, CCRC – Past Investigator
Lisa Zbizek-Nulph, MS, CCRP

Mayo Clinic, Rochester:
Ronald Petersen, MD, PhD
Sara S. Mason, RN
Colleen S. Albers, RN
David Knopman, MD
Kris Johnson, RN

Baylor College of Medicine:
Javier Villanueva-Meyer, MD
Valory Pavlik, PhD
Nathaniel Pacini, MA
Ashley Lamb, MA
Joseph S. Kass, MD, LD, FAAN
Rachelle S. Doody, MD, PhD – Past Investigator
Victoria Shibley, MS – Past Investigator
Munir Chowdhury, MBBS, MS – Past Investigator
Susan Rountree, MD – Past Investigator
Mimi Dang, MD – Past Investigator

Columbia University Medical Center:
Yaakov Stern, PhD
Lawrence S. Honig, MD, PhD
Akiva Mintz, MD, PhD

Washington University, St. Louis:
Beau Ances, MD, PhD, MSc
John C. Morris, MD
David Winkfield, BS
Maria Carroll, RN, MSN, GCNS-BC
Georgia Stobbs-Cucchi, RN, CCRP—Past Investigator
Angela Oliver, RN, BSN, MSG – Past Investigator
Mary L. Creech, RN, MSW – Past Investigator
Mark A. Mintun, MD – Past Investigator
Stacy Schneider, APRN, BC, GNP – Past Investigator

University of Alabama - Birmingham:
David Geldmacher, MD
Marissa Natelson Love, MD
Randall Griffith, PhD, ABPP – Past Investigator
David Clark, MD – Past Investigator
John Brockington, MD – Past Investigator
Daniel Marson, JD, PhD – Past Investigator

Mount Sinai School of Medicine:
Hillel Grossman, MD
Martin A. Goldstein, MD
Jonathan Greenberg, BA
Effie Mitsis, PhD – Past Investigator

Rush University Medical Center:
Raj C. Shah, MD
Melissa Lamar, PhD
Patricia Samuels

Wien Center:
Ranjan Duara, MD
Maria T. Greig-Custo, MD
Rosemarie Rodriguez, PhD

Johns Hopkins University:
Marilyn Albert, PhD
Chiadi Onyike, MD
Leonie Farrington, RN
Scott Rudow, BS
Rottislav Brichko, BS
Stephanie Kielb, BS – Past Investigator

University of South Florida: USF Health Byrd Alzheimer's Institute:
Amanda Smith, MD
Balebail Ashok Raj, MD – Past Investigator
Kristin Fargher, MD – Past Investigator

New York University:
Martin Sadowski, MD, PhD
Thomas Wisniewski, MD
Melanie Shulman, MD
Arline Faustin, MD
Julia Rao, PhD
Karen M. Castro, BA
Anaztasia Ulysse, BA
Shannon Chen, BA
Mohammed O. Sheikh, MD – Past Investigator
Jamika Singleton-Garvin, CCRP – Past Investigator

Duke University Medical Center:
P. Murali Doraiswamy, MBBS, FRCP
Jeffrey R. Petrella, MD
Olga James, MD

Terence Z. Wong, MD
Salvador Borges-Neto, MD – Past Investigator

University of Pennsylvania:
Jason H. Karlawish, MD
David A. Wolk, MD
Sanjeev Vaishnavi, MD
Christopher M. Clark, MD – Past Investigator
Steven E. Arnold, MD – Past Investigator

University of Kentucky:
Charles D. Smith, MD

Gregory A. Jicha, MD, PhD
Riham El Khouli, MD
Flavius D. Raslau, MD

University of Pittsburgh:
Oscar L. Lopez, MD
MaryAnn Oakley, MA
Donna M. Simpson, CRNP, MPH

University of Rochester Medical Center:
Anton P. Porsteinsson, MD
Kim Martin, RN
Nancy Kowalski, MS, RNC
Melanie Keltz, RN
Bonnie S. Goldstein, MS, NP – Past Investigator
Kelly M. Makino, BS – Past Investigator
M. Saleem Ismail, MD – Past Investigator
Connie Brand, RN – Past Investigator

University of California Irvine IMIND:
Gaby Thai, MD
Aimee Pierce, MD
Beatriz Yanez, RN
Elizabeth Sosa, PhD
Megan Witbracht, PhD

University of Texas Southwestern Medical School:
Brendan Kelley, MD
Trung Nguyen, MD
Kyle Womack, MD
Dana Mathews, MD, PhD– Past Investigator
Mary Quiceno, MD– Past Investigator

Emory University:
Allan I. Levey, MD, PhD
James J. Lah, MD, PhD
Ihab Hajjar, MD
Janet S. Cellar, DNP, PMHCNS-BC – Past Investigator

University of Kansas, Medical Center:
Jeffrey M. Burns, MD
Russell H. Swerdlow, MD
William M. Brooks, PhD

University of California, Los Angeles:
Daniel H.S. Silverman, MD, PhD
Sarah Kremen, MD
Liana Apostolova, MD – Past Investigator
Kathleen Tingus, PhD – Past Investigator
Po H. Lu, PsyD – Past Investigator
George Bartzokis, MD – Past Investigator
Ellen Woo, PhD – Past Investigator
Edmond Teng, MD, PhD – Past Investigator

Mayo Clinic, Jacksonville:
Neill R Graff-Radford, MBBCH, FRCP (London)
Francine Parfitt, MSH, CCRC
Kim Poki-Walker, BA

Indiana University:
Martin R. Farlow, MD
Ann Marie Hake, MD – Past Investigator
Brandy R. Matthews, MD – Past Investigator
Jared R. Brosch, MD
Scott Herring, RN, CCRC

Yale University School of Medicine:
Christopher H. van Dyck, MD
Adam P. Mecca, MD, PhD
Adam P. Mecca, MD, PhD
Susan P. Good, APRN
Martha G. MacAvoy, PhD
Richard E. Carson, PhD
Pradeep Varma, MD

McGill Univ., Montreal-Jewish General Hospital:
Howard Chertkow, MD
Susan Vaitekunis, MD
Chris Hosein, MEd

Sunnybrook Health Sciences, Ontario:
Sandra Black, MD, FRCPC
Bojana Stefanovic, PhD
Chris (Chinthaka) Heyn, BSC, PhD, MD, FRCPC

U.B.C. Clinic for AD & Related Disorders:
Ging-Yuek Robin Hsiung, MD, MHSc, FRCPC
Ellen Kim, BA
Benita Mudge, BS
Vesna Sossi, PhD
Howard Feldman, MD, FRCPC – Past Investigator
Michele Assaly, MA – Past Investigator

St. Joseph's Health Care:
Elizabeth Finger, MD
Stephen Pasternak, MD
Irina Rachinsky, MD
Andrew Kertesz, MD – Past Investigator
Dick Drost, MD – Past Investigator
John Rogers, MD – Past Investigator

Northwestern University:
Ian Grant, MD
Brittanie Muse, MSPH
Emily Rogalski, PhD
Jordan Robson
M.-Marsel Mesulam, MD – Past Investigator
Diana Kerwin, MD – Past Investigator
Chuang-Kuo Wu, MD, PhD – Past Investigator
Nancy Johnson, PhD – Past Investigator
Kristine Lipowski, MA– Past Investigator
Sandra Weintraub, PhD – Past Investigator
Borna Bonakdarpour, MD– Past Investigator

Nathan Kline Institute:
Nunzio Pomara, MD
Raymundo Hernando, MD
Antero Sarrael, MD

University of California, San Francisco:
Howard J. Rosen, MD
Bruce L. Miller, MD
David Perry, MD

Georgetown University Medical Center:
Raymond Scott Turner, MD, PhD
Kathleen Johnson, NP
Brigid Reynolds, NP
Kelly MCCann, BA
Jessica Poe, BS

Brigham and Women's Hospital:
Reisa A. Sperling, MD
Keith A. Johnson, MD
Gad A. Marshall, MD

Stanford University:
Jerome Yesavage, MD
Joy L. Taylor, PhD
Steven Chao, MD, PhD
Jaila Coleman, BA
Jessica D. White, BA – Past Investigator
Barton Lane, MD – Past Investigator
Allyson Rosen, PhD – Past Investigator
Jared Tinklenberg, MD – Past Investigator

Banner Sun Health Research Institute:
Christine M. Belden, PsyD
Alireza Atri, MD, PhD
Bryan M. Spann, DO, PhD
Kelly A. Clark
Edward Zamrini, MD – Past Investigator
Marwan Sabbagh, MD – Past Investigator

Boston University:
Ronald Killiany, PhD
Robert Stern, PhD
Jesse Mez, MD, MS
Neil Kowall, MD – Past Investigator
Andrew E. Budson, MD – Past Investigator

Howard University:
Thomas O. Obisesan, MD, MPH
Oyonumo E. Ntekim, MD, PhD
Saba Wolday, MSc
Javed I. Khan, MD
Evaristus Nwulia, MD
Sheeba Nadarajah, PhD

Case Western Reserve University:
Alan Lerner, MD
Paula Ogrocki, PhD
Curtis Tatsuoka, PhD
Parianne Fatica, BA, CCRC

University of California, Davis – Sacramento:
Evan Fletcher, PhD
Pauline Maillard, PhD
John Olichney, MD
Charles DeCarli, MD
Owen Carmichael, PhD – Past Investigator

Dent Neurologic Institute:
Vernice Bates, MD

Horacio Capote, MD
Michelle Rainka, PharmD, CCRP

Parkwood Institute:
Michael Borrie, MB ChB
T-Y Lee, PhD
Dr Rob Bartha, PhD

University of Wisconsin:
Sterling Johnson, PhD
Sanjay Asthana, MD
Cynthia M. Carlsson, MD, MS

Banner Alzheimer's Institute:
Allison Perrin, PhD
Anna Burke, PhD – Past Investigator

Ohio State University:
Douglas W. Scharre, MD
Maria Kataki, MD, PhD
Rawan Tarawneh, MD
Brendan Kelley, MD – Past Investigator

Albany Medical College:
David Hart, MD
Earl A. Zimmerman, MD
Dzintra Celmins, MD

University of Iowa College of Medicine
Delwyn D. Miller, PharmD, MD
Laura L. Boles Ponto, PhD
Karen Ekstam Smith, RN
Hristina Koleva, MD
Hyungsub Shim, MD
Ki Won Nam, MD – Past Investigator
Susan K. Schultz, MD – Past Investigator

Wake Forest University Health Sciences:
Jeff D. Williamson, MD, MHS
Suzanne Craft, PhD
Jo Cleveland, MD
Mia Yang, MD– Past Investigator
Kaycee M. Sink, MD, MAS – Past Investigator

Rhode Island Hospital:
Brian R. Ott, MD
Jonathan Drake, MD
Geoffrey Tremont, PhD
Lori A. Daiello, Pharm.D, ScM
Jonathan D. Drake, MD

Cleveland Clinic Lou Ruvo Center for Brain Health:
Marwan Sabbagh, MD
Aaron Ritter, MD
Charles Bernick, MD, MPH – Past Investigator
Donna Munic, PhD – Past Investigator
Akiva Mintz, MD, PhD – Past Investigator

Roper St. Francis Healthcare:
Abigail O'Connelll, MS, APRN, FNP-C
Jacobo Mintzer, MD, MBA
Arthur Wiliams, BS

Houston Methodist Neurological Institute:
Joseph Masdeu, PhD

Barrow Neurological Institute:
Jiong Shi, MD, PhD
Angelica Garcia, BS
Marwan Sabbagh – Past Investigator

Vanderbilt University Medical Center:
Paul Newhouse, PhD

Long Beach VA Neuropsychiatric Research Program:
Steven Potkin, PhD

Butler Hospital Memory and Aging Program:
Stephen Salloway, MD, MS
Paul Malloy, PhD
Stephen Correia, PhD

Neurological Care of CNY:
Smita Kittur, MD – Past Investigator

Hartford Hospital, Olin Neuropsychiatry Research Center:
Godfrey D. Pearlson, MD – Past Investigator
Karen Blank, MD – Past Investigator
Karen Anderson, RN – Past Investigator

Dartmouth-Hitchcock Medical Center:
Laura A. Flashman, PhD – Past Investigator
Marc Seltzer, MD – Past Investigator
Mary L. Hynes, RN, MPH – Past Investigator
Robert B. Santulli, MD – Past Investigator

Michael Lin, MD – Past Investigator
Lisa Ravdin, PhD – Past Investigator

Cornell University
Norman Relkin, MD, PhD – Past Investigator
Gloria Chiang, MD – Past Investigator
Athena Lee, PhD

II. DOD ADNI

Part A: Leadership and Infrastructure

Principal Investigator

Michael W. Weiner, MD	University of California, San Francisco

ATRI PI and Director of Coordinating Center Clinical Core

Paul Aisen, MD	University of Southern California
Co Director Clinical Core Ron Petersen	Mayo Clinic

Executive Committee

Michael W. Weiner, MD	University of California, San Francisco
Paul Aisen, MD	University of Southern California
Ronald Petersen, MD, PhD	Mayo Clinic, Rochester
Robert C. Green, MD, MPH	Brigham and Women's Hospital/ Harvard Medical School
Danielle Harvey, PhD	University of California, Davis
Clifford R. Jack, Jr., MD	Mayo Clinic, Rochester
William Jagust, MD	University of California, Berkeley
John C. Morris, MD	Washington University St. Louis
Andrew J. Saykin, PsyD	Indiana University
Leslie M. Shaw, PhD	Perelman School of Medicine, University of Pennsylvania
Arthur W. Toga, PhD	University of Southern California
John Q. Trojanowski, MD, PhD	Perelman School of Medicine, University of Pennsylvania

Psychological Evaluation/PTSD Core

Thomas Neylan, MD	University of California, San Francisco

Traumatic Brain Injury/TBI Core

Jordan Grafman, PhD	Rehabilitation Institute of Chicago, Feinberg School of Medicine, Northwestern University

Data and Publication Committee (DPC)

Robert C. Green, MD, MPH	BWH/HMS (Chair)

Resource Allocation Review Committee

Tom Montine, MD, PhD	University of Washington (Chair)

Clinical Core Leaders and Key Personnel

Michael W. Weiner MD	Core PI
Ronald Petersen, MD, PhD	Mayo Clinic, Rochester (Core PI)
Paul Aisen, MD	University of Southern California (Core PI)
Gustavo Jimenez, MBS	University of Southern California
Michael Donohue, PhD	University of Southern California
Devon Gessert, BS	University of Southern California
Jennifer Salazar, MBS	University of Southern California
Caileigh Zimmerman, MS	University of Southern California
Sarah Walter, MSc	University of Southern California
Olusegun Adegoke, MSc	University of Southern California
Payam Mahboubi, MPH	University of Southern California

Lindsey Hergesheimer, BS University of Southern California
Sarah Danowski, MA University of Southern California
Godfrey Coker, MBA, MPH University of Southern California
Taylor Clanton, MPH University of Southern California
Jeremy Pizzola, BA University of Southern California
Elizabeth Shaffer, BS University of Southern California
Catherine Nguyen-Barrera, MS University of Southern California

San Francisco Veterans Affairs Medical Center
Thomas Neylan, MD University of California, San Francisco
Jacqueline Hayes University of California, San Francisco
Shannon Finley University of California, San Francisco

Biostatistics Core Leaders and Key Personnel
Danielle Harvey, PhD University of California, Davis (Core PI)
Michael Donohue, PhD University of California, San Diego

MRI Core Leaders and Key Personnel
Clifford R. Jack, Jr., MD Mayo Clinic, Rochester (Core PI)
Matthew Bernstein, PhD Mayo Clinic, Rochester
Bret Borowski, RT Mayo Clinic
Jeff Gunter, PhD Mayo Clinic
Matt Senjem, MS Mayo Clinic
Kejal Kantarci Mayo Clinic
Chad Ward Mayo Clinic
Duygu Tosun-Turgut, PhD University of California, San Francisco
Stephanie Rossi Chen, BA NCIRE / The Vererans Health Research Institute

PET Core Leaders and Key Personnel
Susan Landau, PhD University of California, Berkeley Core PI

Robert A. Koeppe, PhD University of Michigan
Norm Foster, MD University of Utah
Eric M. Reiman, MD Banner Alzheimer's Institute
Kewei Chen, PhD Banner Alzheimer's Institute

Neuropathology Core Leaders
John C. Morris, MD Washington University St. Louis
Richard J. Perrin, MD, PhD Washington University St. Louis
Erin Franklin, MS Washington University St. Louis

Biomarkers Core Leaders and Key Personnel
Leslie M. Shaw, PhD Perelman School of Medicine, University of Pennsylvania
John Q. Trojanowski, MD, PhD Perelman School of Medicine, University of Pennsylvania
Magdalena Korecka, PhD Perelman School of Medicine, University of Pennsylvania
Michal Figurski, PhD Perelman School of Medicine, University of Pennsylvania

Informatics Core Leaders and Key Personnel
Arthur W. Toga, PhD University of Southern California (Core PI)
Karen Crawford University of Southern California

Scott Neu, PhD University of Southern California

Genetics Core Leaders and Key Personnel

Andrew J. Saykin, PsyD University
Tatiana M. Foroud, PhD Indiana University
Steven Potkin, MD UC UC Irvine
Li Shen, PhD Indiana University

Kelley Faber, MS, CCRC Indiana University
Sungeun Kim, PhD Indiana University
Kwangsik Nho, PhD Indiana University
Kristi Wilmes, MS, CCRP NCRAD

Part B: Investigators By Site

University of Southern California:
Lon S. Schneider, MD
Sonia Pawluczyk, MD
Mauricio Becerra, MD
Liberty Teodoro, RN
Karen Dagerman, MS
Bryan M. Spann, DO, PhD – Past Investigator

University of California, San Diego:
James Brewer, MD, PhD
Helen Vanderswag, RN
Adam Fleisher, MD – Past Investigator

Columbia University Medical Center:
Yaakov Stern, PhD
Lawrence S. Honig, MD, PhD
Akiva Mintz, MD, PhD

Rush University Medical Center:
Raj C. Shah, MD
Ajay Sood, MD, PhD
Kimberly S. Blanchard, DNP, APRN, NP-C
Debra Fleischman, PhD – Past Investigator
Konstantinos Arfanakis, PhD – Past Investigator

Wien Center:
Dr. Ranjan Duara MD PI
Dr. Daniel Varon MD Co-PI
Maria T Greig HP Coordinator

Duke University Medical Center:
P. Murali Doraiswamy, MBBS, FRCP
Jeffrey R. Petrella, MD
Olga James, MD– Past Investigator
Salvador Borges-Neto, MD
Terence Z. Wong, MD

University of Rochester Medical Center:
Anton P. Porsteinsson, MD
Bonnie Goldstein, MS, NP
Kimberly S. Martin, RN

University of California, Irvine:
Gaby Thai, MD
Aimee Pierce, MD
Christopher Reist, MD
Beatriz Yanez, RN
Elizabeth Sosa, PhD
Megan Witbracht, PhD

Premiere Research Inst (Palm Beach Neurology):
Carl Sadowsky, MD
Walter Martinez, MD
Teresa Villena, MD

University of California, San Francisco:
Howard Rosen, MD
David Perry

Georgetown University Medical Center:
Raymond Scott Turner, MD, PhD
Kathleen Johnson, NP
Brigid Reynolds, NP
Kelly MCCann, BA
Jessica Poe, BS

Brigham and Women's Hospital:
Reisa A. Sperling, MD
Keith A. Johnson, MD

Gad Marshall, MD

Banner Sun Health Research Institute:
Christine M. Belden, PsyD
Alireza Atri, MD, PhD
Bryan M. Spann, DO, PhD
Kelly A. Clark
Edward Zamrini, MD – Past Investigator
Marwan Sabbagh, MD – Past Investigator

Howard University:
Thomas O. Obisesan, MD, MPH
Oyonumo E. Ntekim, MD, PhD
Saba Wolday, MSc
Evaristus Nwulia, MD
Sheeba Nadarajah, PhD, RN

University of Wisconsin:
Sterling Johnson, PhD
Sanjay Asthana, MD
Cynthia M. Carlsson, MD, MS

University of Washington:
Elaine R. Peskind, MD
Eric C. Petrie, MD, MS
Gail Li, MD, PhD

Stanford University:
Jerome Yesavage, MD
Joy L. Taylor, PhD
Steven Chao, MD, PhD
Jaila Coleman, BA
Jessica D. White, BA – Past Investigator
Barton Lane, MD – Past Investigator
Allyson Rosen, PhD – Past Investigator
Jared Tinklenberg, MD – Past Investigator

Cornell University:
Michael Lin, PhD
Gloria Chiang, MD
Lisa Ravdin, PhD
Norman Relkin, MD, PhD – Past Investigator

Roper St. Francis Healthcare:
Abigail O'Connelll, MS, APRN, FNP-C
Jacobo Mintzer, MD, MBA
Arthur Wiliams, BS

ADNI Alzheimer's Disease Neuroimaging Initiative

III. ADNI Depression

Part A: Leadership and Infrastructure

Principal Investigator

Scott Mackin, PhD	University of California, San Francisco

ATRI Coordinating Center Clinical Core

Paul Aisen, MD	University of Southern California
Rema Raman, PhD	University of Southern California
Gustavo Jimenez-Maggiora, MBS	University of Southern California
Michael Donohue, PhD	University of Southern California
Devon Gessert, BS	University of Southern California
Jennifer Salazar, MBS	University of Southern California
Caileigh Zimmerman, MS	University of Southern California
Sarah Walter, MSc	University of Southern California
Olusegun Adegoke, MSc	University of Southern California
Payam Mahboubi, MPH	University of Southern California

Executive Committee

Scott Mackin, PhD	University of California, San Francisco
Michael W. Weiner, MD	University of California, San Francisco
Paul Aisen, MD	University of Southern California
Rema Raman, PhD	University of Southern California
Clifford R. Jack, Jr., MD	Mayo Clinic, Rochester
Susan Landau, PhD	University of California, Berkeley
Andrew J. Saykin, PsyD	Indiana University
Arthur W. Toga, PhD	University of Southern California
Charles DeCarli, MD	University of California, Davis
Robert A. Koeppe, PhD	University of Michigan

Data and Publication Committee (DPC)

Robert C. Green, MD, MPH	BWH/HMS (Chair)
Erin Drake, MA	BWM/HMS (Director)

Clinical Core Leaders

Michael W. Weiner MD	Core PI
Paul Aisen, MD	University of Southern California
Rema Raman, PhD	University of Southern California
Mike Donohue, PhD	University of Southern California

Psychiatry Site Leaders and Key Personnel

Scott Mackin, PhD	University of California, San Francisco
Craig Nelson, MD	University of California, San Francisco
David Bickford, BA	University of California, San Francisco
Meryl Butters, PhD	University of Pittsburgh
Michelle Zmuda, MA	University of Pittsburgh

MRI Core Leaders and Key Personnel

Clifford R. Jack, Jr., MD Mayo Clinic, Rochester (Core PI)
Matthew Bernstein, PhD Mayo Clinic, Rochester
Bret Borowski, RT Mayo Clinic, Rochester
Jeff Gunter, PhD Mayo Clinic, Rochester
Matt Senjem, MS Mayo Clinic, Rochester
Kejal Kantarci, MD Mayo Clinic, Rochester
Chad Ward, BA Mayo Clinic, Rochester
Denise Reyes, BS Mayo Clinic, Rochester

PET Core Leaders and Key Personnel
Robert A. Koeppe, PhD University of Michigan
Susan Landau, PhD University of California, Berkeley

Informatics Core Leaders and Key Personnel
Arthur W. Toga, PhD University of Southern California (Core PI)
Karen Crawford University of Southern California
Scott Neu, PhD University of Southern California

Genetics Core Leaders and Key Personnel
Andrew J. Saykin, PsyD Indiana University
Tatiana M. Foroud, PhD Indiana University
Kelley M. Faber, MS, CCRC Indiana University
Kwangsik Nho, PhD Indiana University
Kelly N. Nudelman Indiana University

<u>Part B: Investigators By Site</u>

University of California, San Francisco:
Scott Mackin, PhD
Howard Rosen, MD
Craig Nelson, MD
David Bickford, BA
Yiu Ho Au, BA
Kelly Scherer, BS
Daniel Catalinotto, BA
Samuel Stark, BA
Elise Ong, BA
Dariella Fernandez, BA

University of Pittsburgh:
Meryl Butters, PhD
Michelle Zmuda, BS
Oscar L. Lopez, MD
MaryAnn Oakley, MA
Donna M. Simpson, CRNP, MPH
