# Supplementary material for: MRI-based deep learning can discriminate between temporal lobe epilepsy, Alzheimer’s disease, and healthy controls
Source: Commun Med (Lond). 2023 Feb 27;3:33. doi: 10.1038/s43856-023-00262-4 (PMC9970972; doi:10.1038/s43856-023-00262-4)
Supplement: Supplementary file 4 — Supplementary Data 1 [file 43856_2023_262_MOESM4_ESM.pdf]

Supplementary Data

Properly Trained models					Shuffled models			
Accuracy	Recall	Precision	F1		Accuracy	Recall	Precision	F1
85.40895	0.724754	0.746982	0.734458		67.26513	0.329577	0.266152	0.363744
85.49444	0.730198	0.742834	0.73603		65.98271	0.33012	0.327923	0.279007
86.68187	0.760151	0.778494	0.768465		68.04408	0.333333	0.520661	0.684783
89.43669	0.807421	0.804755	0.805813		67.97758	0.333114	0.389444	0.345382
87.41332	0.776945	0.787387	0.78171		66.71416	0.330268	0.30045	0.262669
88.23027	0.784189	0.798186	0.79056		66.35319	0.331598	0.384764	0.274663
88.17327	0.801211	0.793124	0.79673		67.33162	0.333696	0.309542	0.257079
86.2259	0.725999	0.755788	0.738276		65.94471	0.334987	0.345567	0.292586
87.22333	0.765533	0.775064	0.770032		68.04408	0.333333	0.520661	0.684783
88.20177	0.769912	0.789543	0.778493		66.87565	0.333828	0.308399	0.270361
85.72243	0.737792	0.754572	0.744245		66.84715	0.333297	0.332821	0.268581
86.26389	0.716687	0.753446	0.728058		67.90159	0.334325	0.449548	0.359017
85.80792	0.717011	0.752105	0.729769		68.04408	0.333333	0.520661	0.684783
86.76736	0.725593	0.768535	0.741509		68.04408	0.333333	0.520661	0.684783
85.25696	0.727883	0.744465	0.73334		67.15114	0.332645	0.316483	0.258517
85.31395	0.731885	0.746849	0.736802		64.6718	0.329201	0.334403	0.298241
86.1974	0.732188	0.753186	0.741061		67.24613	0.334542	0.371477	0.260889
88.85722	0.784004	0.789426	0.786633		67.98708	0.334693	0.34568	0.23726
87.6698	0.760441	0.769582	0.764503		64.71929	0.330022	0.331904	0.299058
87.50831	0.754538	0.772038	0.762429		67.7021	0.334579	0.458176	0.247836
88.88572	0.801831	0.813219	0.806981		68.04408	0.333333	0.520661	0.684783
87.6318	0.743423	0.785392	0.758344		66.94215	0.331005	0.31199	0.258646
84.70599	0.716097	0.733339	0.72289		67.96808	0.332761	0.35389	0.343157
86.40638	0.740841	0.77263	0.754075		68.04408	0.333333	0.520661	0.684783
85.23796	0.731874	0.734566	0.733031		67.36012	0.335	0.291109	0.391757
83.76556	0.683728	0.721693	0.697536		66.98015	0.332264	0.330708	0.261646
85.48494	0.72736	0.744739	0.73499		62.91441	0.325353	0.32518	0.310807
88.55324	0.777963	0.807657	0.790732		65.50774	0.33597	0.341977	0.301962
85.35195	0.724067	0.74214	0.732157		67.11314	0.331687	0.419715	0.255395
87.21383	0.769091	0.766603	0.766468		67.97758	0.333272	0.443494	0.346346
86.1594	0.721212	0.743525	0.728813		68.04408	0.333333	0.520661	0.684783
86.05491	0.733637	0.757931	0.744135		68.00608	0.333073	0.360274	0.343008
88.73373	0.769371	0.802487	0.782956		68.04408	0.333333	0.520661	0.684783
87.14734	0.761647	0.774176	0.7672		67.13214	0.332706	0.282863	0.389549
86.26389	0.748504	0.758179	0.752121		65.62173	0.329732	0.334663	0.2848
88.18277	0.746226	0.814182	0.768148		68.04408	0.333333	0.520661	0.684783
87.47981	0.773839	0.787384	0.779524		66.02071	0.331073	0.334884	0.280517
88.23976	0.779335	0.804737	0.788281		68.00608	0.333598	0.436923	0.347079
87.06184	0.754965	0.777774	0.764979		68.04408	0.333333	0.520661	0.684783
87.7458	0.76512	0.789222	0.773835		61.76499	0.330182	0.329288	0.325477
88.83823	0.764977	0.787333	0.774263		67.91109	0.33384	0.430624	0.355139
87.52731	0.761572	0.776791	0.768562		67.81609	0.332508	0.406134	0.351339
86.27339	0.721693	0.757164	0.733215		68.04408	0.333333	0.520661	0.684783
86.1879	0.720354	0.742967	0.727451		68.04408	0.333333	0.520661	0.684783
86.71986	0.76746	0.774701	0.770614		67.80659	0.332994	0.414532	0.355573

85.96941	0.737194	0.749276	0.742805	66.1347	0.337131	0.352948	0.294685
86.97635	0.740323	0.765606	0.750362	67.93958	0.334289	0.384883	0.237147
84.07903	0.704985	0.706916	0.705592	65.60274	0.329354	0.31616	0.284204
86.80536	0.750182	0.772601	0.760022	67.87309	0.334195	0.362771	0.237685
85.38995	0.716493	0.741671	0.726688	66.1822	0.33126	0.295335	0.27828
85.98841	0.725228	0.757373	0.738508	67.11314	0.333119	0.311555	0.261484
86.41588	0.770972	0.761579	0.765507	67.36962	0.331897	0.337711	0.246503
86.68187	0.741261	0.76097	0.749999	67.54061	0.332013	0.414193	0.364429
87.31832	0.742059	0.789477	0.760019	68.04408	0.333333	0.520661	0.684783
86.87185	0.725114	0.765089	0.739391	68.02508	0.333203	0.385265	0.342669
84.649	0.716845	0.721474	0.717561	66.71416	0.334971	0.326234	0.277619
89.67417	0.795072	0.817593	0.804412	67.23663	0.33191	0.371063	0.252691
87.09034	0.780359	0.769084	0.774253	66.65717	0.333167	0.337906	0.273206
86.78636	0.72531	0.779278	0.74489	66.66667	0.334291	0.34582	0.276273
87.10934	0.746527	0.768436	0.755302	68.04408	0.333333	0.520661	0.684783
84.21203	0.689527	0.717586	0.700737	67.86359	0.33396	0.44326	0.358737
87.49881	0.772904	0.77739	0.775087	64.6623	0.330873	0.334461	0.301106
86.78636	0.752773	0.76018	0.755504	68.03458	0.333242	0.260371	0.684788
87.31832	0.746043	0.770959	0.756743	68.04408	0.333333	0.520661	0.684783
86.1119	0.724637	0.736059	0.729842	65.38425	0.333954	0.322267	0.298291
85.66543	0.73729	0.740993	0.738194	66.1157	0.333808	0.347453	0.286235
87.02384	0.767631	0.76671	0.766567	66.43868	0.331399	0.327352	0.272952
82.91061	0.696066	0.717148	0.699508	66.85665	0.330731	0.329462	0.258785
86.42538	0.75096	0.771435	0.759917	67.94908	0.333139	0.316021	0.230568
87.46082	0.763645	0.769103	0.766174	67.27463	0.335108	0.32909	0.263926
86.24489	0.728316	0.756648	0.740144	67.20813	0.33274	0.331353	0.256212
85.74143	0.727367	0.745099	0.734933	66.25819	0.327992	0.308392	0.266249
85.97891	0.712559	0.741696	0.723733	67.19863	0.33207	0.447096	0.254353
89.03771	0.788383	0.81204	0.798887	68.04408	0.333333	0.520661	0.684783
88.68624	0.773955	0.796801	0.783238	68.02508	0.3342	0.456736	0.350399
86.79586	0.74839	0.771014	0.758023	65.70723	0.330923	0.336318	0.286378
85.73193	0.725342	0.747898	0.733773	67.41712	0.332314	0.346306	0.248415
86.35889	0.721203	0.745738	0.730729	66.57167	0.33719	0.38508	0.286551
87.83129	0.764801	0.788747	0.775047	67.30313	0.334088	0.452524	0.258671
86.55837	0.741699	0.761387	0.74981	68.04408	0.333333	0.520661	0.684783
86.46338	0.719985	0.75551	0.733207	67.24613	0.331231	0.273648	0.375395
88.23976	0.7705	0.805836	0.785415	66.1252	0.334268	0.325258	0.287858
84.11703	0.696848	0.715796	0.704118	67.6926	0.334102	0.437469	0.369663
86.89085	0.727417	0.777528	0.744271	68.04408	0.333333	0.520661	0.684783
87.17583	0.756415	0.776333	0.765319	68.03458	0.333347	0.460302	0.343131
83.57557	0.690063	0.711345	0.697158	67.36962	0.331963	0.30211	0.248847
85.76992	0.696869	0.740337	0.708161	66.57167	0.332417	0.331223	0.272948
87.6508	0.738684	0.776038	0.75122	68.04408	0.333543	0.482602	0.344023
87.57481	0.748625	0.789166	0.764549	67.33162	0.332245	0.351603	0.250856
86.53937	0.73768	0.776457	0.753628	66.87565	0.331399	0.320709	0.261716
86.82436	0.721185	0.77358	0.740333	67.94908	0.333979	0.348586	0.234826
86.38738	0.75434	0.753804	0.752902	67.43612	0.337343	0.392563	0.266523

85.96941	0.731518	0.748508	0.738604	68.03458	0.333295	0.426966	0.342731
87.7648	0.752655	0.78673	0.766579	67.26513	0.332027	0.324452	0.252221
87.92628	0.763606	0.782187	0.770199	62.71492	0.335409	0.33652	0.328414
86.48238	0.749692	0.757364	0.753316	67.78759	0.333178	0.419813	0.358037
88.60074	0.78248	0.80361	0.791748	66.47668	0.334893	0.315658	0.28246
89.31319	0.766366	0.806083	0.778864	65.54574	0.332768	0.34366	0.293791
86.84336	0.714616	0.77029	0.732876	67.30313	0.333771	0.426189	0.387523
89.1612	0.776539	0.823743	0.793477	66.65717	0.337523	0.354379	0.286005

Control				TLE			
Accuracy	Recall	Precision	F1	Accuracy	Recall	Precision	F1
79.72357	0.836617	0.787278	0.811198	88.37276	0.806034	0.836315	0.820896
79.60958	0.823207	0.79304	0.807842	89.31319	0.827155	0.84612	0.83653
80.52152	0.844007	0.794641	0.81858	89.58393	0.809052	0.866975	0.837012
85.69393	0.866995	0.859468	0.863215	92.88971	0.873276	0.908113	0.890354
81.88943	0.830597	0.823162	0.826863	89.69792	0.855603	0.836494	0.845941
83.08635	0.848112	0.830608	0.839269	91.00883	0.869397	0.860128	0.864737
83.08635	0.815271	0.853337	0.83387	90.58136	0.890086	0.835694	0.862033
81.43346	0.863437	0.796918	0.828845	89.34169	0.815517	0.855335	0.834951
82.91536	0.846743	0.828824	0.837688	89.2562	0.834914	0.839255	0.837079
83.35708	0.85988	0.827278	0.843264	91.93502	0.878017	0.878017	0.878017
79.33884	0.804324	0.799946	0.802129	88.94272	0.865948	0.812045	0.838131
80.721	0.842638	0.798289	0.819864	90.39612	0.884052	0.835098	0.858878
80.59276	0.846196	0.79445	0.819507	88.68624	0.838362	0.822758	0.830487
81.57595	0.874384	0.793001	0.831706	90.55286	0.838793	0.870694	0.854446
79.41009	0.796388	0.805871	0.801101	87.83129	0.868103	0.786105	0.825072
79.2676	0.79283	0.805841	0.799283	87.94528	0.870259	0.787441	0.826781
81.27672	0.863711	0.794562	0.827695	90.31063	0.797414	0.898058	0.844749
84.36877	0.858238	0.844145	0.851133	93.8444	0.900862	0.911867	0.906331
82.03192	0.835249	0.82242	0.828785	93.30294	0.907759	0.891617	0.899616
82.27415	0.856048	0.813313	0.834133	92.34825	0.866379	0.898525	0.882159
84.15503	0.850848	0.845756	0.848295	90.7381	0.874569	0.849665	0.861937
82.70162	0.869732	0.811542	0.83963	91.17982	0.875862	0.859924	0.86782
78.74038	0.804324	0.790904	0.797558	87.44657	0.835345	0.79524	0.814799
81.60445	0.857964	0.802406	0.829255	87.88829	0.805172	0.82436	0.814653
80.52152	0.809797	0.814927	0.812354	87.6603	0.82931	0.803676	0.816292
76.47478	0.820197	0.75094	0.784042	87.67455	0.804741	0.819219	0.811916
79.25335	0.836344	0.780787	0.807611	90.16814	0.806466	0.88589	0.844314
83.65631	0.874932	0.822485	0.847898	91.39356	0.856466	0.879982	0.868065
79.68082	0.836617	0.78667	0.810875	88.89997	0.803017	0.852632	0.827081
82.06042	0.799945	0.847001	0.822801	90.88059	0.921121	0.823824	0.86976
81.36221	0.835523	0.811968	0.823577	89.9259	0.878879	0.827181	0.852247
80.64976	0.846743	0.794964	0.820037	89.04246	0.81681	0.84636	0.831323

83.96979	0.881773	0.822989	0.851367	92.67598	0.878448	0.897797	0.888017
81.30522	0.828955	0.815124	0.821981	90.99459	0.874569	0.856118	0.865245
82.16016	0.85988	0.809377	0.833864	88.48675	0.77069	0.866279	0.815693
83.14335	0.892173	0.805137	0.846423	91.30806	0.868534	0.868534	0.868534
81.91793	0.827313	0.825731	0.826521	90.0684	0.875431	0.832718	0.853541
83.39983	0.836617	0.84331	0.839951	90.25363	0.905603	0.818784	0.860008
81.87518	0.85249	0.809511	0.830445	89.82616	0.835776	0.853433	0.844512
82.60188	0.83607	0.83084	0.833447	90.66686	0.900862	0.831014	0.864529
84.44001	0.8763	0.83342	0.854322	94.07239	0.909052	0.911409	0.910229
82.73012	0.859332	0.818134	0.838227	91.22257	0.843966	0.885172	0.864078
80.82075	0.840175	0.801148	0.820198	89.9259	0.877586	0.827979	0.852061
80.67826	0.827586	0.8064	0.816856	90.86634	0.899569	0.836473	0.866874
80.721	0.812534	0.816332	0.814429	89.27045	0.858621	0.824162	0.841039
80.37903	0.830597	0.800158	0.815093	89.89741	0.834052	0.856574	0.845163
81.83243	0.849754	0.810493	0.829659	90.90909	0.870259	0.856961	0.863559
78.56939	0.788998	0.79729	0.793122	88.01653	0.842241	0.804446	0.82291
80.89199	0.843459	0.800312	0.821319	90.46737	0.844828	0.863817	0.854217
79.9658	0.83607	0.791041	0.812932	88.51525	0.825862	0.826575	0.826218
80.27928	0.856048	0.784747	0.818848	89.56968	0.812931	0.863553	0.837478
80.97749	0.78763	0.837358	0.811733	88.84298	0.875862	0.804116	0.838457
81.34796	0.850027	0.803207	0.825954	90.85209	0.843103	0.87556	0.859025
81.91793	0.876847	0.796421	0.834701	90.55286	0.838793	0.870694	0.854446
81.71844	0.868637	0.798089	0.83187	91.03733	0.860345	0.867449	0.863882
78.17042	0.776683	0.798537	0.787458	89.02821	0.885345	0.802971	0.842148
85.22371	0.871374	0.84884	0.859959	93.24594	0.914224	0.885225	0.899491
81.76119	0.80104	0.841092	0.820578	90.31063	0.881034	0.834967	0.857383
81.37646	0.885605	0.784485	0.831984	90.19664	0.818103	0.877079	0.846566
81.86093	0.843459	0.814697	0.828829	91.05158	0.878879	0.854568	0.866553
78.29866	0.835249	0.768185	0.800315	88.00228	0.796552	0.833183	0.814456
82.00342	0.835523	0.821803	0.828606	91.5218	0.8625	0.878788	0.870568
81.84668	0.820197	0.829275	0.824711	90.15389	0.884483	0.829091	0.855892
82.2599	0.874384	0.802562	0.836935	92.26275	0.834052	0.92451	0.876954
80.7495	0.834975	0.803106	0.818731	91.97777	0.867672	0.887175	0.877315
79.75207	0.794198	0.812658	0.803322	89.64092	0.88319	0.817964	0.849326
81.5617	0.831965	0.817204	0.824519	91.73554	0.843534	0.900184	0.870939
77.04474	0.840449	0.749207	0.792209	86.07865	0.6625	0.887926	0.758825
80.87774	0.855227	0.79355	0.823235	89.04246	0.7875	0.868759	0.826136
82.43089	0.845101	0.82237	0.833581	92.3625	0.8625	0.902164	0.881886
80.73525	0.85468	0.791836	0.822058	90.18239	0.82931	0.867839	0.848138
79.78056	0.825397	0.794311	0.809556	89.89741	0.85	0.845264	0.847625
80.33628	0.850848	0.788286	0.818373	90.96609	0.853879	0.870387	0.862054
84.48276	0.877121	0.83355	0.854781	91.93502	0.866379	0.887026	0.876581
83.65631	0.859606	0.832053	0.845605	93.0037	0.908621	0.883117	0.895687
81.67569	0.845649	0.810598	0.827752	89.54118	0.843966	0.840343	0.842151
80.10829	0.822386	0.800906	0.811504	89.01396	0.860345	0.817028	0.838127
81.14848	0.84948	0.800619	0.824326	91.15133	0.86681	0.865691	0.86625
83.15759	0.860974	0.82356	0.841852	90.58136	0.857759	0.857389	0.857574

81.20547	0.835249	0.809764	0.822309
81.21972	0.863164	0.794058	0.82717
82.82987	0.8763	0.809608	0.841635
78.28441	0.807882	0.782194	0.79483
81.11998	0.864532	0.791928	0.826639
82.00342	0.861248	0.806303	0.83287
76.77401	0.786809	0.771605	0.779133
78.93987	0.840175	0.77447	0.805986
82.67313	0.864806	0.814013	0.838641
82.57338	0.876574	0.805786	0.839691
80.35053	0.867542	0.779828	0.82135
81.43346	0.89272	0.781692	0.833525
81.16272	0.796114	0.834481	0.814846
80.23653	0.825397	0.801062	0.813048
82.57338	0.87329	0.807644	0.839185
83.59932	0.842638	0.842408	0.842523
81.13423	0.82786	0.813172	0.82045
83.65631	0.861522	0.830826	0.845895
84.82474	0.8763	0.839318	0.857411
81.09148	0.88451	0.781242	0.829675
84.78199	0.881773	0.835148	0.857827

90.02565	0.862069	0.840336	0.851064
90.72385	0.850431	0.866491	0.858386
91.43631	0.846121	0.889443	0.867241
87.31832	0.826724	0.797174	0.81168
90.83785	0.864655	0.859101	0.861869
90.66686	0.823707	0.885953	0.853697
87.00484	0.84181	0.781825	0.81071
91.65004	0.896983	0.857084	0.87658
92.09176	0.899138	0.866639	0.882589
90.7096	0.844397	0.870667	0.85733
90.25363	0.80431	0.890267	0.845109
91.00883	0.81681	0.901951	0.857272
89.68367	0.896983	0.81099	0.851822
89.9259	0.858621	0.840152	0.849286
91.72129	0.856897	0.88869	0.872504
90.98034	0.906034	0.835121	0.869134
90.21089	0.851293	0.852395	0.851844
91.72129	0.876724	0.873336	0.875027
93.95839	0.944828	0.881029	0.911814
91.70704	0.851293	0.892857	0.87158
91.73554	0.896121	0.859801	0.877585

		AD	
Accuracy	Recall	Precision	F1
88.13052	0.531609	0.617353	0.571282
87.56056	0.54023	0.589342	0.563718
89.94015	0.627395	0.673868	0.649802
89.72642	0.681992	0.646685	0.663869
90.65261	0.644636	0.702505	0.672328
90.59561	0.635057	0.703822	0.667674
90.85209	0.698276	0.690341	0.694286
87.90254	0.499042	0.615112	0.551031
89.49843	0.614943	0.657114	0.635329
89.31319	0.571839	0.663333	0.614198
88.88572	0.543103	0.651724	0.592476
87.67455	0.423372	0.62695	0.505432
88.14477	0.466475	0.639108	0.539313
88.17327	0.463602	0.64191	0.538376
88.5295	0.519157	0.64142	0.573849
88.72898	0.532567	0.647264	0.584341
87.00484	0.535441	0.566937	0.550739
88.35851	0.592912	0.612265	0.602433

87.67455	0.538314	0.594709	0.565108
87.90254	0.541188	0.604278	0.570995
91.76404	0.680077	0.744235	0.710711
89.01396	0.484674	0.684709	0.567583
87.93103	0.508621	0.613873	0.556312
89.72642	0.559387	0.691124	0.618317
87.53206	0.556513	0.585096	0.570447
87.14734	0.426245	0.59492	0.496652
87.03334	0.539272	0.56754	0.553045
90.60986	0.60249	0.720504	0.656234
87.47506	0.532567	0.587117	0.558513
88.70048	0.586207	0.628983	0.606842
87.19008	0.449234	0.591425	0.510615
88.4725	0.537356	0.632469	0.581046
89.55543	0.547893	0.686675	0.609483
89.14221	0.581418	0.651288	0.614372
88.14477	0.614943	0.598881	0.606805
90.09689	0.477969	0.768875	0.589486
90.45312	0.618774	0.703704	0.658512
91.06583	0.595785	0.752116	0.664885
89.48418	0.576628	0.670379	0.619979
89.96865	0.558429	0.705811	0.623529
88.00228	0.509579	0.617169	0.558237
88.62924	0.581418	0.627066	0.60338
88.07353	0.447318	0.642366	0.527386
87.01909	0.433908	0.586028	0.498624
90.16814	0.631226	0.68361	0.656375
87.6318	0.546935	0.591097	0.568159
88.18752	0.500958	0.629362	0.557867
85.65118	0.483716	0.519013	0.500744
89.05671	0.562261	0.653675	0.604531
87.6888	0.487548	0.607399	0.540914
88.11627	0.506705	0.623821	0.559197
89.42719	0.649425	0.643264	0.64633
87.84554	0.530651	0.604144	0.565018
89.48418	0.510536	0.701316	0.590909
87.85979	0.44636	0.62973	0.522422
86.74836	0.488506	0.562914	0.523077
90.55286	0.599617	0.718714	0.653786
89.1992	0.659004	0.631193	0.644799
88.78598	0.472222	0.676269	0.55612
88.4155	0.517241	0.636042	0.570523
86.33514	0.436782	0.551391	0.48744
88.97122	0.62069	0.631579	0.626087
88.35851	0.55364	0.622174	0.58591
87.43232	0.529693	0.585805	0.556338
85.60844	0.471264	0.517895	0.49348

87.60331	0.534483	0.592357	0.561934
87.77429	0.627395	0.58274	0.604244
85.60844	0.585249	0.51431	0.547491
89.35594	0.610153	0.651996	0.630381
87.58906	0.583333	0.582775	0.583054
87.81704	0.500958	0.610268	0.550237
87.54631	0.506705	0.595721	0.547619
86.63437	0.43295	0.566416	0.490771
90.69535	0.621648	0.715546	0.6653
89.39869	0.55364	0.675234	0.608421
89.1707	0.555556	0.6621	0.604167
88.07353	0.493295	0.625759	0.551687
86.77686	0.447318	0.570905	0.501611
89.75492	0.57567	0.685291	0.625716
88.444	0.527778	0.634062	0.576059
87.44657	0.44636	0.605982	0.514065
90.45312	0.58908	0.718458	0.647368
86.74836	0.455939	0.568019	0.505845
88.71473	0.453065	0.681556	0.544304
88.85722	0.584291	0.636743	0.609391
86.94785	0.441571	0.580605	0.501632
86.71986	0.353448	0.589457	0.441916
88.18752	0.452107	0.647462	0.532431
89.44144	0.524904	0.691047	0.596625
89.01396	0.541188	0.659277	0.594424
88.03078	0.454023	0.637097	0.530201
88.31576	0.569923	0.615942	0.59204
87.7458	0.510536	0.604308	0.553479
88.99972	0.527778	0.663855	0.588047
89.1992	0.542146	0.669031	0.598942
88.10202	0.569923	0.606524	0.587654
90.42462	0.609195	0.706667	0.654321
89.15645	0.477969	0.697902	0.567368
87.73155	0.408046	0.636771	0.497373
90.96609	0.551724	0.77628	0.645017

Control		TLE		AD	
Mean	Structure	Mean	Structure	Mean	Structure
0.32	Calcarine_R	0.433	Hippocampus_R	0.401	Hippocampus_R
0.299	Vermis_10	0.315	Vermis_3	0.364	Hippocampus_L
0.28	Angular_L	0.303	Calcarine_R	0.334	SupraMarginal_L
0.277	SupraMarginal_L	0.293	Angular_L	0.326	Angular_L
0.271	Occipital_Mid_L	0.266	Hippocampus_L	0.32	Occipital_Mid_L
0.255	Caudate_R	0.263	Pallidum_L	0.287	Angular_R
0.253	Angular_R	0.26	Amygdala_L	0.268	Caudate_L
0.249	Occipital_Sup_L	0.246	Thalamus_L	0.262	Occipital_Sup_L
0.228	Occipital_Sup_R	0.244	Vermis_10	0.252	Caudate_R
0.21	Frontal_Inf_Tri_L	0.24	Occipital_Mid_L	0.245	Parietal_Inf_L
0.206	Frontal_Inf_Oper_L	0.229	Thalamus_R	0.225	Temporal_Inf_L
0.203	Frontal_Inf_Tri_R	0.215	Caudate_L	0.215	SupraMarginal_R
0.198	Frontal_Mid_R	0.206	ParaHippocampal_R	0.215	Occipital_Sup_R
0.194	Occipital_Mid_R	0.204	Angular_R	0.214	Occipital_Inf_L
0.191	SupraMarginal_R	0.196	Amygdala_R	0.206	Vermis_10
0.185	Occipital_Inf_L	0.178	Caudate_R	0.204	Temporal_Mid_L
0.178	Frontal_Inf_Oper_R	0.174	Frontal_Mid_L	0.202	Parietal_Inf_R
0.174	Frontal_Mid_L	0.169	Occipital_Mid_R	0.198	Occipital_Mid_R
0.165	Vermis_9	0.163	SupraMarginal_L	0.194	Frontal_Mid_R
0.164	Calcarine_L	0.155	Frontal_Inf_Tri_L	0.183	Calcarine_R
0.163	Hippocampus_R	0.153	Frontal_Inf_Oper_L	0.173	Temporal_Inf_R
0.162	Caudate_L	0.151	Pallidum_R	0.17	Temporal_Mid_R
0.151	Frontal_Sup_Orb_R	0.147	Calcarine_L	0.164	Occipital_Inf_R
0.149	Frontal_Sup_R	0.14	Cingulum_Post_R	0.154	Parietal_Sup_L
0.139	Parietal_Inf_L	0.13	Frontal_Mid_R	0.153	Frontal_Mid_L
0.137	Precentral_L	0.128	Temporal_Inf_L	0.15	Postcentral_L
0.132	Cuneus_R	0.128	Fusiform_R	0.149	Precentral_L
0.131	Cingulum_Post_R	0.128	Parietal_Inf_L	0.146	Precentral_R
0.128	Frontal_Sup_Orb_L	0.121	Occipital_Sup_R	0.145	Frontal_Inf_Tri_R
0.124	Frontal_Sup_L	0.117	Cerebelum_Crus1_L	0.137	Frontal_Inf_Oper_L
0.12	Putamen_R	0.114	Frontal_Inf_Tri_R	0.133	Lingual_L
0.118	Rolandic_Oper_L	0.112	SupraMarginal_R	0.128	Frontal_Inf_Tri_L
0.113	Parietal_Inf_R	0.105	Precentral_L	0.123	Calcarine_L
0.109	Temporal_Mid_L	0.102	Frontal_Sup_R	0.118	Frontal_Inf_Oper_R

0.108	Lingual_R	0.102	Putamen_L	0.115	Postcentral_R
0.107	Putamen_L	0.101	ParaHippocampal_L	0.114	Rolandic_Oper_L
0.107	Lingual_L	0.1	Occipital_Inf_L	0.113	Lingual_R
0.106	Temporal_Inf_L	0.099	Temporal_Mid_L	0.113	Frontal_Sup_R
0.106	Temporal_Mid_R	0.099	Temporal_Mid_R	0.107	Vermis_9
0.103	Postcentral_L	0.098	Frontal_Sup_L	0.102	Pallidum_R
0.103	Parietal_Sup_L	0.096	Lingual_R	0.097	Pallidum_L
0.098	Precentral_R	0.092	Cerebelum_3_L	0.094	Insula_L
0.097	Rolandic_Oper_R	0.091	Parietal_Inf_R	0.094	Frontal_Sup_L
0.088	Occipital_Inf_R	0.09	Frontal_Inf_Oper_R	0.091	Cuneus_R
0.088	Thalamus_L	0.088	Lingual_L	0.087	Cuneus_L
0.087	Postcentral_R	0.085	Temporal_Inf_R	0.08	Precuneus_R
0.087	Insula_L	0.081	Cingulum_Post_L	0.079	Precuneus_L
0.082	Cuneus_L	0.08	Parietal_Sup_L	0.072	Fusiform_R
0.081	Temporal_Inf_R	0.078	Vermis_9	0.069	Fusiform_L
0.08	Frontal_Mid_Orb_L	0.066	Frontal_Sup_Orb_L	0.058	Frontal_Sup_Orb_R
0.067	Precuneus_R	0.065	Postcentral_L	0.055	Thalamus_L
0.065	Thalamus_R	0.064	Cerebelum_Crus1_R	0.055	Putamen_L
0.064	Frontal_Mid_Orb_R	0.061	Cerebelum_Crus2_L	0.051	Rolandic_Oper_R
0.062	Fusiform_R	0.06	Cerebelum_6_R	0.05	Cingulum_Ant_R
0.059	Pallidum_R	0.059	Occipital_Sup_L	0.05	Thalamus_R
0.048	Vermis_4_5	0.056	Putamen_R	0.049	Cingulum_Ant_L
0.047	Fusiform_L	0.051	Precuneus_R	0.046	Frontal_Sup_Orb_L
0.046	Hippocampus_L	0.049	Postcentral_R	0.042	Frontal_Mid_Orb_L
0.042	Cingulum_Post_L	0.049	Occipital_Inf_R	0.042	Vermis_4_5
0.041	Precuneus_L	0.046	Precentral_R	0.041	Cingulum_Post_L
0.039	Cingulum_Ant_L	0.042	Cuneus_R	0.04	Parietal_Sup_R
0.039	Insula_R	0.042	Fusiform_L	0.038	Cingulum_Post_R
0.035	Vermis_8	0.041	Frontal_Mid_Orb_L	0.037	Olfactory_L
0.034	Pallidum_L	0.037	Cerebelum_6_L	0.036	Temporal_Sup_L
0.034	Parietal_Sup_R	0.036	Cingulum_Ant_R	0.035	ParaHippocampal_L
0.033	Temporal_Sup_L	0.033	Vermis_7	0.034	Temporal_Pole_Sup_L
0.033	Frontal_Inf_Orb_L	0.03	Insula_L	0.029	Frontal_Mid_Orb_R
0.032	Frontal_Med_Orb_R	0.029	Precuneus_L	0.028	Temporal_Sup_R
0.031	Rectus_R	0.029	Parietal_Sup_R	0.028	Putamen_R
0.03	Cingulum_Ant_R	0.027	Vermis_4_5	0.028	Olfactory_R
0.028	Temporal_Sup_R	0.025	Cingulum_Ant_L	0.024	ParaHippocampal_R
0.026	Cerebelum_3_L	0.025	Frontal_Sup_Orb_R	0.02	Insula_R
0.024	Frontal_Inf_Orb_R	0.025	Cerebelum_Crus2_R	0.019	Cerebelum_Crus2_L
0.023	Cerebelum_Crus2_L	0.023	Supp_Motor_Area_R	0.019	Vermis_8
0.022	Supp_Motor_Area_R	0.017	Olfactory_L	0.019	Frontal_Inf_Orb_L
0.021	Olfactory_L	0.017	Cuneus_L	0.01	Cerebelum_Crus2_R
0.021	Olfactory_R	0.017	Cerebelum_9_L	0.01	Cerebelum_Crus1_R
0.019	Rectus_L	0.016	Frontal_Sup_Medial_[illegible]	0.01	Supp_Motor_Area_R
0.017	Supp_Motor_Area_L	0.016	Insula_R	0.009	Rectus_R
0.015	Cerebelum_Crus1_R	0.015	Olfactory_R	0.009	Temporal_Pole_Mid_R
0.011	Cerebelum_Crus2_R	0.015	Cerebelum_3_R	0.008	Temporal_Pole_Sup_R

0.01	Frontal_Sup_Medial_	0.013	Cerebelum_4_5_L	0.008	Cerebelum_3_L
0.01	Frontal_Sup_Medial_	0.013	Frontal_Sup_Medial_	0.007	Cingulum_Mid_L
0.009	Frontal_Med_Orb_L	0.011	Vermis_6	0.006	Supp_Motor_Area_L
0.007	Temporal_Pole_Sup_	0.011	Paracentral_Lobule_R	0.005	Cingulum_Mid_R
0.007	Paracentral_Lobule_R	0.01	Rectus_L	0.005	Frontal_Inf_Orb_R
0.005	Cerebelum_Crus1_L	0.008	Rolandic_Oper_L	0.005	Vermis_7
0.005	Cerebelum_9_L	0.007	Supp_Motor_Area_L	0.004	Frontal_Sup_Medial_R
0.005	Vermis_7	0.006	Temporal_Pole_Sup_	0.003	Rectus_L
0.004	Heschl_L	0.006	Frontal_Mid_Orb_R	0.003	Cerebelum_Crus1_L
0.003	ParaHippocampal_L	0.005	Rolandic_Oper_R	1.00E-03	Amygdala_R
0.002	ParaHippocampal_R	0.005	Temporal_Sup_R	1.00E-03	Frontal_Med_Orb_R
0.002	Cerebelum_9_R	0.004	Frontal_Inf_Orb_L	1.00E-03	Cerebelum_6_R
1.00E-03	Cerebelum_6_R	0.004	Frontal_Inf_Orb_R	1.00E-03	Cerebelum_4_5_L
0	Temporal_Pole_Mid_	0.004	Cerebelum_9_R	0	Paracentral_Lobule_L
0	Temporal_Pole_Sup_	0.004	Cerebelum_4_5_R	0	Heschl_R
0	Temporal_Pole_Mid_	0.003	Frontal_Med_Orb_R	0	Amygdala_L
0	Paracentral_Lobule_L	0.003	Cingulum_Mid_L	0	Heschl_L
0	Cerebelum_3_R	0.002	Cingulum_Mid_R	0	Frontal_Sup_Medial_L
0	Cerebelum_7b_L	1.00E-03	Temporal_Sup_L	0	Cerebelum_6_L
0	Cerebelum_4_5_R	1.00E-03	Temporal_Pole_Sup_	0	Cerebelum_3_R
0	Cerebelum_6_L	0	Cerebelum_7b_L	0	Cerebelum_4_5_R
0	Cerebelum_4_5_L	0	Temporal_Pole_Mid_	0	Temporal_Pole_Mid_L
0	Cerebelum_7b_R	0	Temporal_Pole_Mid_	0	Cerebelum_7b_L
0	Cerebelum_8_L	0	Cerebelum_8_R	0	Cerebelum_7b_R
0	Vermis_1_2	0	Cerebelum_8_L	0	Cerebelum_9_L
0	Amygdala_L	0	Cerebelum_7b_R	0	Cerebelum_10_R
0	Cerebelum_10_L	0	Cerebelum_10_L	0	Cerebelum_8_R
0	Cerebelum_10_R	0	Heschl_L	0	Vermis_1_2
0	Cingulum_Mid_L	0	Rectus_R	0	Cerebelum_9_R
0	Amygdala_R	0	Cerebelum_10_R	0	Vermis_3
0	Cerebelum_8_R	0	Vermis_1_2	0	Paracentral_Lobule_R
0	Heschl_R	0	Vermis_8	0	Frontal_Med_Orb_L
0	Cingulum_Mid_R	0	Heschl_R	0	Cerebelum_8_L
0	Vermis_6	0	Frontal_Med_Orb_L	0	Cerebelum_10_L
0	Vermis_3	0	Paracentral_Lobule_L	0	Vermis_6
