# Supplementary material for: MRI-based deep learning can discriminate between temporal lobe epilepsy, Alzheimer’s disease, and healthy controls
Source: Commun Med (Lond). 2023 Feb 27;3:33. doi: 10.1038/s43856-023-00262-4 (PMC9970972; doi:10.1038/s43856-023-00262-4)
Supplement: Supplementary file 5 — Reporting Summary [file 43856_2023_262_MOESM5_ESM.pdf]

nature portfolio

Corresponding author(s): *Double-blind peer review submissions: write DBPR and your manuscript number here instead of author names.*

Last updated by author(s): *YYYY-MM-DD*

Reporting Summary

Nature Portfolio wishes to improve the reproducibility of the work that we publish. This form provides structure for consistency and transparency in reporting. For further information on Nature Portfolio policies, see our Editorial Policies and the Editorial Policy Checklist.

Statistics

For all statistical analyses, confirm that the following items are present in the figure legend, table legend, main text, or Methods section.

n/a	Confirmed	
☐	☒	The exact sample size (n) for each experimental group/condition, given as a discrete number and unit of measurement
☐	☒	A statement on whether measurements were taken from distinct samples or whether the same sample was measured repeatedly
☐	☒	The statistical test(s) used AND whether they are one- or two-sided<br>*Only common tests should be described solely by name; describe more complex techniques in the Methods section.*
☐	☒	A description of all covariates tested
☐	☒	A description of any assumptions or corrections, such as tests of normality and adjustment for multiple comparisons
☐	☒	A full description of the statistical parameters including central tendency (e.g. means) or other basic estimates (e.g. regression coefficient) AND variation (e.g. standard deviation) or associated estimates of uncertainty (e.g. confidence intervals)
☒	☐	For null hypothesis testing, the test statistic (e.g. F, t, r) with confidence intervals, effect sizes, degrees of freedom and P value noted<br>*Give P values as exact values whenever suitable.*
☒	☐	For Bayesian analysis, information on the choice of priors and Markov chain Monte Carlo settings
☒	☐	For hierarchical and complex designs, identification of the appropriate level for tests and full reporting of outcomes
☒	☐	Estimates of effect sizes (e.g. Cohen's d, Pearson's r), indicating how they were calculated

Our web collection on statistics for biologists contains articles on many of the points above.

Software and code

Policy information about availability of computer code

Data collection	N/A- Multicenter data collection, various software and scanners
Data analysis	MATLAB R2021a, SPM12(7771), CAT12.8.1

For manuscripts utilizing custom algorithms or software that are central to the research but not yet described in published literature, software must be made available to editors and reviewers. We strongly encourage code deposition in a community repository (e.g. GitHub). See the Nature Portfolio guidelines for submitting code & software for further information.

Data

Policy information about availability of data

All manuscripts must include a data availability statement. This statement should provide the following information, where applicable:

- Accession codes, unique identifiers, or web links for publicly available datasets
- A description of any restrictions on data availability
- For clinical datasets or third party data, please ensure that the statement adheres to our policy

The datasets generated during and/or analysed during the current study are available from the corresponding author on reasonable request.

Human research participants

Policy information about studies involving human research participants and Sex and Gender in Research.

Reporting on sex and gender	There is no gender or sex based analysis. Sex is reported in the demographic table. Gender is not.
Population characteristics	Age is displayed in the demographic table
Recruitment	Data was taken from multiple databases collected from various sites. Temporal lobe epilepsy patients and their healthy controls were collected from the Medical University of South Carolina, Emory University, and University of Bonn. The Alzheimer's disease patients and their healthy controls were taken from the publicly available database ADNI. Details are further describe in section 2.1 .
Ethics oversight	The internal review board (IRB) for each site it was collected.

Note that full information on the approval of the study protocol must also be provided in the manuscript.

Field-specific reporting

Please select the one below that is the best fit for your research. If you are not sure, read the appropriate sections before making your selection.

☒ Life sciences ☐ Behavioural & social sciences ☐ Ecological, evolutionary & environmental sciences

For a reference copy of the document with all sections, see nature.com/documents/nr-reporting-summary-flat.pdf

Life sciences study design

All studies must disclose on these points even when the disclosure is negative.

Sample size	Sample size was based on the size of the databases.
Data exclusions	Data was excluded if diagnosis was not clear or T1-weighted images were missing or corrupted
Replication	100 replicated models were created for both shuffled and non-shuffled models.
Randomization	Randomization was performed using MATLAB's built-in function: dividerand
Blinding	Software blinded the researchers from which subjects were allocated to training, validation, and testing sets.

Reporting for specific materials, systems and methods

We require information from authors about some types of materials, experimental systems and methods used in many studies. Here, indicate whether each material, system or method listed is relevant to your study. If you are not sure if a list item applies to your research, read the appropriate section before selecting a response.

Materials & experimental systems

n/a	Involved in the study	
☒	☐	Antibodies
☒	☐	Eukaryotic cell lines
☒	☐	Palaeontology and archaeology
☒	☐	Animals and other organisms
☐	☒	Clinical data
☒	☐	Dual use research of concern

Methods

n/a	Involved in the study	
☒	☐	ChIP-seq
☒	☐	Flow cytometry
☐	☒	MRI-based neuroimaging

Clinical data

Policy information about clinical studies

All manuscripts should comply with the ICMJE guidelines for publication of clinical research and a completed CONSORT checklist must be included with all submissions.

Clinical trial registration	n/a

Study protocol	n/a
Data collection	Participants with TLE and their matched healthy controls were derived from three different sites: The Medical University of South Carolina (Charleston, SC, USA), Emory University (Atlanta, GA, USA), and The University of Bonn (Bonn, Germany). Patients were recruited sequentially between March 2017 and December 2020. ADNI subjects were taken from the ADNI database which details can be found on their website (adni.loni.usc.edu)
Outcomes	n/a

Magnetic resonance imaging

Experimental design

Design type	Resting state
Design specifications	n/a
Behavioral performance measures	n/a

Acquisition

Imaging type(s)	Structural
Field strength	3T
Sequence & imaging parameters	MUSC: Siemens Skyra 3T scanner, isotropic voxel size 1mm, 12-channel head coil, TR = 2050-2250 ms, TE = 2.5-18 ms, FOV = 256- 320 mm, flip angle 10°. Emory: Siemens Prisma 3T scanner, isotropic voxel size 0.8mm, 12-channel head coil, TR = 2300ms, TE = 2.75ms, TI = 1100ms, flip angle 8. Bonn: Siemens Magnetom Trio 3T scanner, 8-channel head coil, isotropic voxel size of 1mm, TR = 650ms, TE = 3.97ms, TI = 650ms, flip angle 10°.
Area of acquisition	Whole Brain

Diffusion MRI ☐ Used ☐ Not used

Preprocessing

Preprocessing software	MATLAB, SPM, CAT12
Normalization	We normalized all T1-weighed images into standard stereotaxic MNI152 space (113x137x113) using the normalize function from the software package Statistical Parametric Mapping (SPM) with the following parameters: bias regularization = 0.0001, bias FWHM = 60, tissue probability map = TPM.nii, voxel size = 1x1x1 mm3, and 4th degree b-spline interpolation.
Normalization template	TPM.nii --> SPM
Noise and artifact removal	n/a
Volume censoring	n/a

Statistical modeling & inference

Model type and settings	Mass univariate
Effect(s) tested	T1-image intensity

Specify type of analysis: ☒ Whole brain ☐ ROI-based ☐ Both

Statistic type for inference (See Eklund et al. 2016)	Voxel-wise
Correction	bonferroni corrected t-test

Models & analysis

n/a	Involved in the study
☒	☐ Functional and/or effective connectivity
☒	☐ Graph analysis
☐	☒ Multivariate modeling or predictive analysis

Multivariate modeling and predictive analysis	T1-image intensity

Multivariate modeling and predictive analysis	Model evaluation --> Accuracy, F1-Score, Precision, Recall
